# Social consequences of planned relocation in response to sea level rise: impacts on anxiety, well-being, and perceived safety

**DOI:** 10.1038/s41598-024-53277-9

**Published:** 2024-02-12

**Authors:** Mumuni Abu, Stacey C Heath, W. Neil Adger, Samuel Nii Ardey Codjoe, Catherine Butler, Tara Quinn

**Affiliations:** 1 Regional Institute for Population Studies, University of Ghana, Legon Boundary, Accra, Ghana; 2 School of Psychology, The Open University, Walton Hall, Milton Keynes, MK7 6AA UK; 3 Geography, Faculty of Environment, Science and Economy, University of Exeter, Exeter, EX4 4RJ UK; 4 Irish Climate Analysis and Research Units (ICARUS), Department of Geography, Maynooth University, Maynooth, Co. Kildare Ireland

**Keywords:** Climate-change adaptation, Psychology and behaviour

## Abstract

Governments globally are adapting to sea level rise through a range of interventions to improve everyday lives of communities at risk. One prominent response is planned relocation, where people and communities are enabled to move from localities exposed to coastal erosion and inundation as a result of sea level rise. Managed retreat has significant social consequences including under-reported impacts on health, well-being and social identity. Here we adopt well-established measures of well-being and document the outcomes of planned relocation on well-being in the Volta Delta region of Ghana. Data from a bespoke survey for individuals (n = 505) in relocated and non-relocated communities demonstrate that planned relocation negatively impacts well-being and anxiety of those relocated when compared to a community that is equally exposed but has not moved. Individuals in the relocated community reported significantly lower levels of overall wellbeing, significantly higher levels of anxiety, and lower perceptions of safety, compared to non-relocated community members. These outcomes are explained as being related to the disruption of community connection, identities, and feelings of efficacy. Relocated community members reported significantly lower levels of attachment to the local area and home, significantly lower levels of community-based self-efficacy, and significantly lower levels of overall community-based identity. The results demonstrate that planned relocation to address sea level rise has multiple social consequences with outcomes for well-being that are not straightforwardly related to risk reduction.

## Introduction

There is considerable uncertainty as to the scale of sea level rise projected for the coming decades and century. The stakes could not be higher for settlements and populations living close to or below sea level, with various estimates indicating between five hundred million and one billion people living in places subject to increasing and more severe flood risk^1^. Commonly, large scale actions to adapt to these risks are ultimately assumed to involve populations migrating away from coastal areas. But moving in such circumstances requires individuals to have agency and the means to make such decisions about their future life and livelihood. Yet many individuals living in risky coastal environments are either unaware of risks or, more commonly, have limited resources or agency to move their place of residence. In this context, government collective interventions for assisting communities to relocate are increasingly common and appear likely to become more so in the future. These interventions, often referred to as managed retreat or planned relocation, involve actions to facilitate movement of whole or partial settlements through land purchase, compensation, and provision of other resources^2^. The consequences of such interventions are, however, not well documented with little evidence on their wider implications for well-being beyond the assumed benefits derived from the avoidance of risk.

Sea level rise has potentially transformative economic and societal consequences on coastal communities and infrastructure, ranging from low lying agricultural areas, through to tourism infrastructure and ports. The associated climatic changes in coastal areas, such as storm surges, groundwater and soil salinity, and changing weather patterns, amplify the consequences of sea level change. The impacts of these processes on places and economies is well understood but the consequences of the outcomes are less well known. Common coastal adaptations include, at their most general, reducing risks through hard engineering of coasts, designing strategies to manage and live with risk, and relocating people and infrastructure away from hazardous places. Engineering and other adaptations have themselves well-documented consequences, including macro-economic costs, impacts on long term economic productivity, and consequences for climate justice on marginalised populations^3^.

There is now growing evidence that focusses specifically on the consequences of planned relocation—moving communities away from exposed coastal localities due to sea level rise and associated environmental risks. That evidence comes from detailed long-term studies in specific localities, and a small number of synthesis studies looking across these cases from diverse parts of the world^4^. Some of this work is framed around the processes of actually moving settlements—how people are enabled, listened to, and empowered to undertake to take ownership of those decisions. Some of this work shows that best practice is rarely implemented and that many communities are marginalised or have in some instances even been coerced into moving. These studies also largely agree that a lack of agency among those being moved leads to negative social outcomes^5^. Some studies examine the fate of communities who have been moved in terms of their livelihood and economic prospects, again commonly finding that communities are not better off in their places of destination. Despite this growing understanding, much of the evidence to date does not directly address how the process of relocating communities makes those involved feel. That is to say, there is very little research examining the implications for psychosocial well-being^6^.

To enhance understanding of the overall impacts of adaptation interventions, it is therefore necessary to incorporate psychosocial dimensions directly into planning strategies and into adaptation evaluation measures. Psychosocial impacts encompass emotional, mental, and social dimensions that arise from changes to social structures and group dynamics^7^ and, importantly, can vary from one community to another. These dimensions have been well established in social psychology and other models of well-being. There are diverse ways of measuring and observing each of these elements. Environmental changes and disruptions, particularly where change is imposed, can lead to psychosocial consequences such as solastalgia, eco-anxiety, psychological distress, and eco-grief, which themselves can have effects on health and well-being outcomes^8–10^. Such consequences can occur concurrently with events, but can also persist long after the environmental disruption has occurred, often with severe consequences for mental health that can be long-lasting and enduring^11^. Inevitably, such psychosocial stressors are often amplified for those populations that are the most vulnerable to the impacts of climate change, such as those with pre-existing mental health difficulties, or those populations facing limited resources, infrastructural deficiencies, health inequalities and socio-political challenges^12^.

Where there has been research on this aspect of relocation, existing findings show that relocation as a part of managed retreat can be traumatic for those involved. There are psychological consequences, mainly negative, of losing both homes and the neighbourhood relations associated with them^13^. Furthermore, there can be significant consequences for cultural heritage, often implicit in stories, narratives, and other cultural expressions, that are impacted by the upheaval of moving home and settlement. Each of these consequences have been documented in specific cases using social and psychological measures of place change such as place attachment indicators and identity^14^. The collective and relational dimensions of such changes are manifest in group identity issues and, importantly, the outcomes of dislocation and displacement can be manifest in reduced well-being or exacerbated in symptoms of mental ill-health (as documented for evacuees from flood events)^15^.

Planned relocation from sea level change appears, at first glance, a rational response to heightened risk and can be evaluated alongside other potential responses including large scale coastal protection projects. Some estimates of the scale of managed retreat suggest there have been a minimum of 1.3 million people relocated in such schemes globally in the past three decades^16^. There are around 20–30 million people being displaced temporarily from weather related disasters globally per year on average over the past decades^17^. The study presented here, therefore, seeks to contribute to the emerging evidence on the implications of planned relocation by examining a real-world example of the psychosocial consequences for coastal communities and the factors that shape them.

We undertook a bespoke social psychological survey in two communities in the Volta Delta region of Ghana, Keta and Totope, that are exposed to the consequences of sea level rise and related erosion. Keta was subject to a planned relocation intervention starting in 2003, while Totope is subject to erosion risk but no one has yet been relocated. Both communities are located in an exposed coastal delta area in eastern Ghana. The Volta delta is a sedimentary fault-controlled basin in the Gulf of Guinea in West Africa, with the whole delta region located within Ghana and a population of close to one million reported in the most recent census. It is characterised by two major lagoons and extensive creeks, mangroves, and marsh areas, and has been subject to sedimentary starvation through dam building and hence currently experiences rates of around 4 mm yr^-1^ sea level rise, exacerbated by aquifer mining and other land use changes^18^, leading to erosion along the coasts and lagoons. Derby and colleagues conclude that erosion is the dominant coastal process in the delta at present, and that the consequences are likely to be greater in incoming decades: ‘flooding and submergence becomes more likely as sea levels rise and subsistence continues’ (p.116)^19^. Scenarios of land use change in the delta by Brempong and colleagues (using a range of RCPs) estimate that > 20% of land in the coastal districts of the Volta region could be below sea level by the end of the century^20^.

The Ghanaian government response, principally to erosion and flooding in the most recent decades, has been to enable planned relocation in the most exposed localities such as Keta. Other localities, such as Totope, have yet to be relocated or enabled to undertake radical adaptations, and are instead anticipating this change and preparing for what relocation may entail. Working with these two different locations, in this study we use a natural experiment of relocated and non-relocated communities to examine the psychosocial consequences of this emerging adaptation strategy.

## Materials, methods and research design

**Figure 1:**
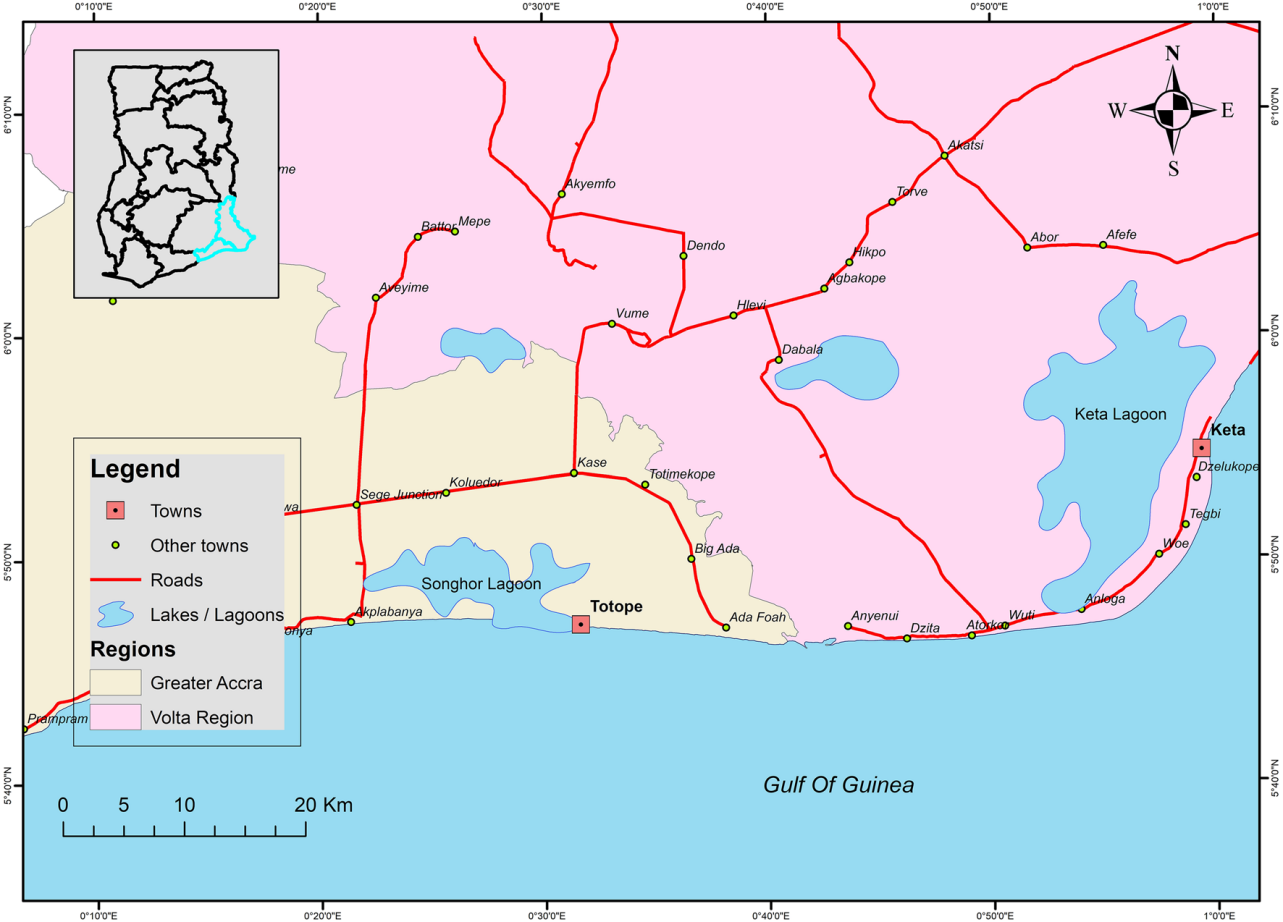
Location of Keta and Totope, Volta region, Ghana. Totope population is reliant on fisheries and have not been relocated due to no suitable relocation site and lack of public funding. Keta community implemented a sea defence scheme with significant populations being moved and protected. Map produced in ArcGIS using ArcMap Version 10.3.

The coastline of Ghana has experienced erosion and flooding for decades due to the impacts of climate change^21^. As a result, the government of Ghana in 2003 embarked on a programme of relocation to reduce risks to lives and property in some of the communities most affected by tidal waves. In the Keta municipal area, shown in Fig. 1, we collected data on the outcomes of some of these government-led interventions for inhabitants in Kedzi and Havedzi where the community has relocated after the construction of the Keta Sea defence wall. The second site is Totope in the Ada East district which is a coastal village located on a long strip of sandy land between the Songhor lagoon and the sea (Fig. 1). It is increasingly exposed to flooding on the coast and in the Songhor Lagoon associated with surges from the Atlantic ocean coinciding with high tides. Residents report that almost half of the land area of the coastal communities has been lost to the ocean over the past two decades. There have been plans by the government to relocate the people of Totope, but this has not happened partly due to local perceptions that the planned relocation site does not have the needed facilities to support the village’s livelihood activities, which are predominantly related to access to the coast and seas, such as fishing. Thus, community members prefer to continue to live in the current location, and have requested that the government intervene with the construction of a sea defence wall.

Both the studied communities have been experiencing high out migration, and both are predominantly fishing communities, with some community members also engaged in agricultural activities. The decision to invest in planned relocation for any exposed coastal community has both economic and political dimensions. Often populations living in such places are marginalised by decisions not to invest while other economic interests and actors prevail in such decisions^22,23^.

The study employed a survey design to investigate the psychosocial determinants of health in the context of communities facing sea-level rise and planned relocation. The sample consisted of participants (n = 505; 58.9% female) from Keta and Totope. The survey measured several key psychosocial variables to comprehensively assess impacts on the overall well-being of the affected populations (see Table 1). The survey instrument used established scales and measures from psychology and social science where available to optimise validity and reliability. For example, social identity measures capture a sense of cohesion and identification with community. Place and home attachment measures developed by social psychologists reflect both the physical place but also cognitive and emotional positive responses^24–27^. Similarly, standard World Health Organisation measures of well-being and generalised anxiety were adopted, all of which have been routinely tested in multiple countries and cultural contexts.

**Table 1:** Key variables in analysis of wellbeing outcomes and mediators in survey of populations subject to managed retreat in Volta region, Ghana.

Key variable	Included to assess	Example survey statements and questions
Social identity	Participants’ sense of cohesion and identification with their community. Included to measure how managed retreat influences the collective dimensions of large-scale group-based changes and how these might impact the relational dynamics within the community^24^	I identify as a member of my community
Attachment to place and home	Strength of emotional bonds that evolve between people and their place of residence or other important settings that are manifest in processes, cognition and physical dimensions associated with one’s attachment to their local area and home^25^. These measures of attachment identify the ways in which managed retreat and relocation might impact the intricate and multifaceted nature of individuals’ attachment to their environment^26,27^	I feel very attached to the local area where I live I feel very attached to my home
Community-based self-efficacy	Individuals’ beliefs in their community’s ability to address challenges and navigate changes. Measures how individuals perceive their community’s capacity to adapt and manage the consequences of sea-level rise, contributing to the broader evaluation of adaptation^28^	How confident are you that your community can accomplish its flood management related goals?
Safety	Participants’ subjective assessment of safety in their community. Uncovering variations in individuals’ perceived safety provides insights into the emotional and cognitive dimensions of relocation. Bespoke questions for this survey	When thinking about flood management activities, I feel safe in my community
Well-being	Participants state of health and life satisfaction: to capture individuals’ holistic sense of wellbeing, incorporating emotional, physical and social dimensions. Ultimately, generates a comprehensive evaluation of the psychological impact of managed retreat on quality of life. Uses WHO Quality of Life scales	Overall, how do you rate your life satisfaction
Anxiety	Participants levels of anxiety and worry: provides insights into the psychological consequences of relocation on mental health. Generalised Anxiety Disorder scale	When thinking about flood management activities how nervous, anxious or on edge do you feel?

The selection of these variables aimed to capture a comprehensive understanding of the psychosocial dimensions (that is, perceptions of safety, community-based self-efficacy, attachment, well-being and feelings of anxiety) that may be impacted by the process of planned relocation. Statistical analyses, including descriptive statistics and inferential tests, were applied to the survey data to discern significant differences between the relocated and non-relocated communities across the measured variables.

The two study communities were sampled because of their histories with flood hazards and relocation. Keta has received significant intervention, including the sea defence construction and resettlement of the people of Havedzi and Kedzi, while the people of Totope have not been relocated but continue to face the impacts of sea level rise. The survey was conducted face to face in December 2021, and received prior ethical approvals from the University of Exeter, Faculty of Environment, Science and Economy ethics committee, and the College of Humanities ethics committee at the University of Ghana. The data collection therefore complied with all relevant regulations and ethical research best practice including informed consent from each participant. We first listed all structures in the selected communities and used a simple random sampling technique to select individuals aged 18 years and older from a household in a structure and carried out the survey with them in-person. A maximum of two households were selected from a structure, and an individual was selected from a household. Table 2 presents a summary of background information of the study participants.

**Table 2:** Percentage distribution of background information of study population.

Variables	Keta (*N* = 252)	Totope (*N* = 253)
Identified gender		
Female	60.3	58.9
Male	39.7	41.1
Age range		
18–30	19.4	40.7
30–45	32.5	31.6
45–60	26.6	19.4
60+	21.4	8.3
Marital status		
Single	11.5	25.3
Living with partner	3.6	15.8
Married	61.1	46.6
Divorced	8.3	4.3
Widowed	15.5	7.9
Education level attained		
None at all	20.2	32.4
Primary school	28.2	24.9
Lower secondary school	32.1	20.9
High school	14.3	15
College	3.6	2
Graduate/professional degree	1.6	4.7
Housing tenure		
Rent privately	6.3	5.1
Rent social housing	1.2	1.6
Owner occupier	50	44.3
Living with family/friends	40.5	49
Holiday home/non-permanent residence	2	0
Length of residence in community		
< 1 year	0.8	2.8
1–5 years	8.7	8.3
6–10 years	5.2	7.9
> 10 years	85.3	81
Current annual income range		
< GH₵10,000	71.4	71.5
GH₵10,000–GH₵20,000	12.3	15.4
> GH₵20,000	16.3	13

More than 50% of the respondents from both communities (Keta—60.3% and Totope—58.9%) are females, which is consistent with the national statistics of female representation in the study areas. While only 19% of the respondents in Keta are between the ages of 18 and 30, a little over two-fifth (41%) of the respondents in Totope are in that age category. Additionally, the percentage of respondents aged 60 years and older is higher in Keta (21.4%) compared to Totope (8.3%). In addition, a higher proportion (60.1%) of the population in Keta is married, compared to 46.6% in Totope. Similarly, the proportion of respondents who are widowed is higher in Keta (15.5%) compared to Totope (7.9%). In terms of education, the proportion of the population with secondary or higher education is higher in Totope (21.7%) than in Keta (19.5%). Also, Totope has a higher proportion of respondents without any formal education (32.4%) compared to Keta (20.2%). The majority of the respondents live in their own houses or are living with friends and family relations, and they have lived in their respective communities for over a decade. The income distribution in the two study communities are similar, with about seven out of ten respondents from each of the study communities living in households with an average annual income of less than GH₵10, 000 at the time of the survey where US$1 was equivalent to GH₵6.25. This translates into an average daily household income of less than US$5 a day, an equivalent of GH₵31.25, which has higher than the daily minimum wage of GH₵13.53.

## Results

The survey results highlight that those community members from relocated communities reported lower levels of well-being across multiple measures, compared to those from non-relocated communities. While it might be anticipated that those who have experienced a government-led intervention reducing their exposure to risk and sea level rise would have increased well-being, this research shows the reverse. The results show that individuals in the non-relocated community of Totope reported significantly higher levels of well-being, perceived safety, community-based self-efficacy, social identity, and attachments to their home and environment compared to those in the relocated area of Keta. In addition, the population in Totope also reported significantly lower levels of anxiety compared to those in Keta: see Table 3 for test statistics, medians (Md), p-values, and interquartile ranges. Thus, contrary to expectations, our findings suggest that populations in relocated communities of Keta feel more insecure, less happy, and have less attachment to their home and the place in general, and their health and well-being is lower than that of the community members in the non-relocated area of Totope.

**Table 3:** Mann–Whitney U test statistics, medians, interquartile ranges, and P-values for all variables between Keta and Totope.

	Keta (*n* = 252), *Md* (*IQR*)	Totope (*n* = 253), *Md* (*IQR*)	*U*	*z*	*p*
Wellbeing	44.17 (14.1)	47.57 (16)	26,931.00	−3.02	0.003
Anxiety	4.15 (2.5)	2.99 (3.5)	23,999.00	−4.88	< 0.001
Community-based self-efficacy	6.34 (4.25)	7.92 (5.5)	23,979.00	−4.83	< 0.001
Perceived safety	4.96 (3.67)	8.37 (3.33)	17,732.50	−8.65	< 0.001
Community-based identity	9.65 (0)	9.71 (1)	28,770.50	−1.94	0.05
Attachment to place	3.93 (0)	4.19 (1)	26,874.50	−3.41	< 0.001
Attachment to home	4.11 (1)	4.28 (1)	27,770.50	−2.83	0.005

We systematically assess a diverse range of psychosocial dimensions that are often overlooked or indirectly addressed across the literature. The primary outcomes from this study highlight the significant social consequences of interventions aimed at mitigating risk to lives and property in communities vulnerable to sea level rise.

The research highlights significant health and well-being consequences manifest in outcomes such as perceived safety, identity, and community-based self-efficacy. Critically, for the coastal communities of Keta and Totope, there are significant differences between relocated and non-relocated places. The relocated community of Keta, strategically moved away from flood risk, reported significantly lower levels of well-being and heightened levels of anxiety, compared to the non-relocated community of Totope. Moreover, the research highlights the broader impact of the relocation process on psychosocial dimensions at both individual and community-levels. This influence on perceptions of safety, efficacy, attachment to place and identity, we suggest, likely contribute to the reduction in well-being and increased levels of anxiety reported. These findings demonstrate how negative outcomes in terms of psychosocial dimensions can arise through and within processes of response to sea level rise, such as relocation.

## Discussion

The results from this study resonate with a growing body of evidence that demonstrate comparable adverse social consequences from relocation interventions. These include economic outcomes such as increased indebtedness and loss of livelihood options, through to social outcomes such as disrupted community ties and a loss of identity^28–32^. The findings clearly demonstrate impacts on socio-psychological well-being in ways that work to disentangle these adverse consequences arising out of adaptation responses from the negative effects of exposure to sea level rise. We show how communities exposed to sea level rise where relocation and government-led adaptation processes have been implemented can have lower reported health and well-being than those in communities without interventions. It is, however, important to examine the ways that the specific processes and approaches deployed for adaptation in this context shaped these outcomes. The implications from this analysis is not to avoid undertaking relocations where necessary, but to more deeply reflect on how interventions that involve moving people and their place of residence intersect with social and economic factors to create outcomes.

The detrimental health and well-being outcomes for those living in communities within Keta compared to Totope, where no intervention has been implemented, can be explained through four main factors related to the intervention: constrained livelihoods, perceptions of effectiveness of the government actions, perceptions of safety, and perceptions of the planning process.

The first concerns the ways that livelihoods are reshaped through adaptation processes. In Keta, the sea defence wall was constructed without paying detailed attention to the ways it could impact on the livelihoods of local people and communities. The interventions focused on land reclamation without considering other socio-cultural and economic issues that collectively define the habitability of a place. The impact of sea level rise led to the loss of landing sites for fishing, which contributed to the initial outmigration from the area. It was expected that any intervention by the government, for example the construction of a fishing harbour in the area, would enhance and re-establish this livelihood opportunity. But no replacement fishing harbour has been built, resulting in more outmigration.

The second factor relates to the ways the interventions are perceived by those affected populations. Again in Keta, the population was very confident that the construction of the sea defence wall would completely address the annual sea flooding they experience in the area, but this has not been the case. Every year, when the tides are high, communities like Kedzi and Havedzi continue to experience sea flooding, a situation that constantly reminds them of the vulnerabilities of the area. Third, the places selected for relocation can heighten experiences of loss. In Keta, members of these communities still live close to the ocean and the area in which they have relocated from, constantly triggering memories of what they have lost to the sea. One consequence of the practice of planned relocation that includes engineered sea defences is the reduction of risk of periodic flooding while not alleviating entirely the risk of catastrophic flooding: a phenomenon referred to as the ‘levee effect’ by Hino and colleagues^16^.

The results here also show that the residents of Keta have greater levels of perceived lack of safety than those in Totope, which remains less protected. This may reflect the perception of long run and still present catastrophic risk, associated with an understanding that sea level change creates an ongoing existential threat to these communities. Sea defence structures designed for present but not future risk can lock in the intervention strategy, and protecting the population behind the hard engineering defence becomes the only viable, but ultimately limited, adaptation option^33^. In the case of Keta, though flooding may be less frequent owing to the sea defence, its consequences are likely to be even greater when it does happen. This means the sense of vulnerability, anxiety, and fear can remain and even be worsened by processes that to some extent reduce, but ultimately do not address, the risks people face.

Continuing proximity to the ocean means that losses as well as anxieties and risk are ever present. The participants report that sight of the ocean alone, can evoke negative feelings because, even with the defence wall interventions, the risk still lingers, making people feel unsafe in their homes. Losses to the sea that they have experienced, such as their ancestral homes and graves, and cultural and religious relics including sacred groves, are also ever present and underpin a weakened attachment to place with further implications for well-being.

Fourth, the nature of the planning processes and their implementation can deeply affect relations of trust and people’s sense of security. In Keta, the response from the government to emergency situations has been primarily reactive, and the population has completely lost hope in disaster management authorities to be able address the situation. There is no wider comprehensive disaster prevention programme, leaving populations in these areas living in very vulnerable conditions, with relocation options not, it appears, being a straightforward solution. The case here demonstrate that rather than reducing vulnerability in an absolute sense, much risk and indeed social vulnerability is redistributed and shifted into other domains of life.

By contrast, stakeholders in Totope have in essence resisted the calls for managed retreat and relocation, at least in the terms on which it has been offered. It has been widely demonstrated that many communities faced with climate risk choose not to spontaneously migrate and also resist planned relocation. This has been referred to as voluntary immobility—people want to stay in places exposed to risk even when faced with deep uncertainty^34^. This study points to the reasons for such voluntary immobility—in essence place and home attachment, perceived well-being and group identity have all been maintained and in some ways, to an extent, strengthened by the non-relocated community. This finding gives impetus to careful planning for managed retreat to ensure these multiple dimensions of social consequences are well integrated into plans before implementation.

The implications of these findings, then, are to highlight the centrality of social, economic and psychological dimensions of the experiences of sea level rise and processes of response. Technical and engineered designs for adaptation to sea level rise are unlikely to achieve desired outcomes for health and well-being without giving focus to the ways such processes intersect with such factors. For example, the livelihoods of populations in coastal communities are critical to building resilience to coastal hazards. Relocation may help reduce the vulnerability of human populations to hazards, but it cannot completely solve other social and economic problems that may arise as a result of, or be exascerbated by relocation.

The results demonstrate that relocating populations without attentiveness to wider social, economic and psychological dimensions may not engender improved outcomes for health and well-being. The population of the Keta communities were predominantly fishers, and they had a major fishing market along the beach that attracted other business activities to the area. The relocation of infrastructure and housing that paved the way for the construction of the Keta sea defences did not factor the business opportunities in the area into the plan. As a result, the relocated population lost their main livelihood, and they perceive that livelihood and overall well-being has been diminshed since the construction of the sea defence wall. In addition, they believed that the construction of the sea defence wall would eliminate frequent flooding of the communities. Yet Kedzi and Havedzi localities flood every year during high tides in August and September: the flooding problem has not been resolved.

Indeed, in the cases examined here, key aspects of life crucial to well-being have been detrimentally affected by processes of adaptation more so than by sea level rise alone. The Keta communities are showing higher levels of anxiety and less attachment to their home and community. Moreover, the construction of the sea defences without adequate provisions for the livelihood options of the people, has created a major economic gap in the Keta community compared to the Totope community. In effect the design of the relocation process and its lack of effectiveness of reducing flood risk in Keta, potentially explains the observed differences in well-being measures. These outcomes highlight the crucial need to integrate understanding of, and thinking about, factors such as livelihoods, community relations, and attachments to place and home in the development and implementation of adaptation processes.

There is good evidence from studies elsewhere that length of residence in a place increases most measures of place attachment which themselves are correlated with positive well-being outcomes^35–37^. The relocation of communities in Keta began in 2003, and it would be likely that place attachment will increase over time. However, such an increase in place attachment would only likely be realised if the recurrent flooding that results from high tides every year and the economic gap in the area is addressed. The Coastal Development Authority through the Government of Ghana is working toward the construction of a fishing harbour in Keta to rejuvenate the economic life of the area and alleviate reductions to economic and well-being outcomes.

This study demonstrates that there are no easy and straightforward adaptations to sea level rise. Any managed retreat involves difficult choices and comes at a cost to aspects of the natural environment and to different groups of people. The imposed harm from human-induced sea level rise, whether through global change or through starving deltas of sediments, constitutes an issue of climate justice. All scenarios of sea level rise for the incoming decades and beyond suggest that the demands and necessity for managed retreat will only increase. However, simply imposing managed retreat as a single solution to the needs for coastal adaptation in itself, runs the risk of unforeseen and negative outcomes for marginalised populations^38^. Hence there is a critical requirement to incorporate multiple insights into how populations at the sharp end perceive and react to the traumatic experience of being relocated, and to maximise the social benefits from the planning, design and justice perspectives.

## Data Availability

The survey data are available from MA on request.

## References

[1] Neumann B, Vafeidis AT, Zimmermann J, Nicholls RJ (2015). Future coastal population growth and exposure to sea-level rise and coastal flooding—A global assessment. PLoS ONE.

[2] Mach KJ, Siders AR (2021). Reframing strategic, managed retreat for transformative climate adaptation. Science.

[3] Thacker S, Adshead D, Fay M, Hallegatte S, Harvey M, Meller H, O’Regan N, Rozenberg J, Watkins G, Hall JW (2019). Infrastructure for sustainable development. Nat. Sustain..

[4] Bower ER, Badamikar A, Wong-Parodi G, Field CB (2023). Enabling pathways for sustainable livelihoods in planned relocation. Nat. Clim. Change.

[5] Ajibade I, Sullivan M, Lower C, Yarina L, Reilly A (2022). Are managed retreat programs successful and just? A global mapping of success typologies, justice dimensions, and trade-offs. Glob. Environ. Change.

[6] Quinn T, Heath S, Adger WN, Abu M, Butler C, Codjoe SNA, Horvath C, Martinez-Juarez P, Morrissey K, Murphy C, Smith R (2023). Health and wellbeing implications of adaptation to flood risk. Ambio.

[7] Heath SC, Rabinovich A, Barreto M (2017). Putting identity into the community: Exploring the social dynamics of urban regeneration. Eur. J. Soc. Psych..

[8] Albrecht G, Sartore GM, Connor L, Higginbotham N, Freeman S, Kelly B, Stain H, Tonna A, Pollard G (2007). Solastalgia: The distress caused by environmental change. Aus. Psychiatry.

[9] Cunsolo A, Ellis NR (2018). Ecological grief as a mental health response to climate change-related loss. Nat. Clim. Change.

[10] Phillips C, Murphy C (2021). Solastalgia, place attachment and disruption: Insights from a coastal community on the front line. Reg. Environ. Change.

[11] Norris FH, Murphy AD, Baker CK, Perilla JL (2004). Postdisaster PTSD over four waves of a panel study of Mexico's 1999 flood. J. Traumatic Stress.

[12] Levy B, Patz J (2015). Climate Change and Public Health.

[13] Binder SB, Baker CK, Barile JP (2015). Rebuild or relocate? Resilience and postdisaster decision-making after Hurricane Sandy. Am. J. Commun. Psych..

[14] Scannell L, Gifford R (2017). The experienced psychological benefits of place attachment. J. Environ. Psych..

[15] Munro A, Kovats RS, Rubin GJ, Waite TD, Bone A, Armstrong B, Beck CR, Amlôt R, Leonardi G, Oliver I (2017). Effect of evacuation and displacement on the association between flooding and mental health outcomes: A cross-sectional analysis of UK survey data. Lancet Plan Health.

[16] Hino M, Field CB, Mach KJ (2017). Managed retreat as a response to natural hazard risk. Nat. Clim. Change.

[17] *Internal Displacement Monitoring Centre, Global Report on Internal Displacement 2023*. https://www.internal-displacement.org/global-report/grid2023/ (IDMC, , 2023).

[18] Codjoe, S.N.A. *et al. *The volta delta, Ghana: Challenges in an African setting. In *Deltas in the Anthropocene* (Nicholls, R. *et al*. eds.). 79–102 (Palgrave, 2020).

[19] Darby, S.E., Appeaning Addo, K., Hazra, S., Rahman, M.M. & Nicholls, R.J. Fluvial sediment supply and relative sea-level rise. In *Deltas in the Anthropocene* (Nicholls, R. *et al*. eds.). 103–126 (Palgrave, 2020).

[20] Brempong EK, Almar R, Angnuureng DB, Mattah PAD, Avornyo SY, Jayson-Quashigah PN, Addo KA, Minderhoud P, Teatini P (2023). Future flooding of the Volta Delta caused by sea level rise and land subsidence. J. Coastal Conserv..

[21] Appeaning Addo K, Larbi L, Amisigo B, Ofori-Danson PK (2011). Impacts of coastal inundation due to climate change in a cluster of urban coastal communities in Ghana, West Africa. Remote Sens..

[22] Owusu-Daaku KN, Rosko H (2019). The discursive construction of adaptation subjects via the Ada Sea Defense System in the Volta River Delta of Ghana. Environ. Plan E Nat. Sp..

[23] Mortreux C, de Campos RS, Adger WN, Ghosh T, Das S, Adams H, Hazra S (2018). Political economy of planned relocation: A model of action and inaction in government responses. Glob. Environ. Change.

[24] Barnett J, Graham S, Quinn T, Adger WN, Butler C (2021). Three ways social identity shapes climate change adaptation. Environ. Res. Lett..

[25] Scannell L, Gifford R (2010). Defining place attachment: A tripartite organizing framework. J. Environ. Psych..

[26] Lewicka M (2011). On the varieties of people’s relationships with places: Hummon’s typology revisited. Environ. Behav..

[27] Boley BB, Strzelecka M, Yeager EP, Ribeiro MA, Aleshinloye KD, Woosnam KM, Mimbs BP (2021). Measuring place attachment with the abbreviated place attachment scale (APAS). J. Environ. Psych..

[28] Kita SM (2017). Urban vulnerability, disaster risk reduction and resettlement in Mzuzu city, Malawi. Int. J. Disaster Risk Reduct..

[29] Arnall A (2019). Resettlement as climate change adaptation: what can be learned from state-led relocation in rural Africa and Asia?. Clim. Dev..

[30] Piggott-McKellar AE, Pearson J, McNamara KE, Nunn PD (2020). A livelihood analysis of resettlement outcomes: Lessons for climate-induced relocations. Ambio.

[31] Ajibade I (2019). Planned retreat in Global South megacities: Disentangling policy, practice, and environmental justice. Clim. Change.

[32] Bergmann J (2021). Planned relocation in Peru: Advancing from well-meant legislation to good practice. J. Environ. Stud. Sci..

[33] Hinkel J, Aerts JC, Brown S, Jiménez JA, Lincke D, Nicholls RJ, Scussolini P, Sanchez-Arcilla A, Vafeidis A, Addo KA (2018). The ability of societies to adapt to twenty-first-century sea-level rise. Nat. Clim. Change.

[34] Farbotko C, Dun O, Thornton F, McNamara KE, McMichael C (2020). Relocation planning must address voluntary immobility. Nat. Clim. Change.

[35] Albers T, Ariccio S, Weiss LA, Dessi F, Bonaiuto M (2021). The role of place attachment in promoting refugees’ well-being and resettlement: A literature review. Int. J. Environ. Res. Public Health.

[36] Scannell L, Gifford R (2017). Place attachment enhances psychological need satisfaction. Environ. Behav..

[37] Swapan MSH, Sadeque S (2021). Place attachment in natural hazard-prone areas and decision to relocate: Research review and agenda for developing countries. Int. J. Dis. Risk Reduct..

[38] Rahman MF, Lewis D, Kuhl L, Baldwin A, Ruszczyk H, Nadiruzzaman M, Mahid Y (2023). Managed urban retreat: The trouble with crisis narratives. Urban Geogr..

